# Mixed Manganese Dioxide on Magnetite Core MnO_2_@Fe_3_O_4_ as a Filler in a High-Performance Magnetic Alginate Membrane

**DOI:** 10.3390/ma14247667

**Published:** 2021-12-12

**Authors:** Paweł Grzybek, Roman Turczyn, Gabriela Dudek

**Affiliations:** Department of Physical Chemistry and Technology of Polymers, Faculty of Chemistry, Silesian University of Technology, Strzody 9, 44-100 Gliwice, Poland; pawegrz205@student.polsl.pl (P.G.); Roman.Turczyn@polsl.pl (R.T.)

**Keywords:** composite membranes, sodium alginate, ethanol dehydration, manganese dioxide, magnetite

## Abstract

The process of ethanol dehydration via pervaporation was performed using alginate membranes filled with manganese dioxide and a mixed filler consisting of manganese dioxide on magnetite core MnO_2_@Fe_3_O_4_ particles. The crystallization of manganese dioxide on magnetite nanoparticle surface resulted in a better dispersibility of this mixed filler in polymer matrix, with the preservation of the magnetic properties of magnetite. The prepared membranes were characterized by contact angle, degree of swelling and SEM microscopy measurements and correlated with their effectiveness in the pervaporative dehydration of ethanol. The results show a strong relation between filler properties and separation efficiency. The membranes filled with the mixed filler outperformed the membranes containing only neat oxide, exhibiting both higher flux and separation factor. The performance changed depending on filler content; thus, the presence of optimum filler loading was observed for the studied membranes. The best results were obtained for the alginate membrane filled with 7 wt.% of mixed filler MnO_2_@Fe_3_O_4_ particles. For this membrane, the separation factor and flux equalled to 483 and 1.22 kg·m^−2^·h^−1^, respectively.

## 1. Introduction

The on-going development in the fields of biofuels and fine chemistry has led to the increase in demand for pure, anhydrous organic solvents, particularly ethanol. Due to the versatility and widespread use of high-purity ethanol, there is a growing interest in the improvement of production methods allowing costs to be reduced and efficiency to be increased [1]. Distillation is a traditional process for the production of ethanol and its major advantage is high alcohol recovery (96%) [2]. Nevertheless, it cannot be used to produce completely dry ethanol and this drawback is associated with the formation of water–ethanol azeotrope. For this reason, alternative methods of ethanol formation have recently been explored, including gas stripping, flash fermentation, vacuum fermentation, liquid-liquid extraction and pervaporation (PV). The latter process, PV, is particularly interesting, mainly because of its simple operation, as well as its lower energy consumption and higher separation efficiency than distillation [3].

The most important part of any PV dehydration unit is a polymeric membrane, for instance, one made of hydrophilic poly(vinyl alcohol), sodium alginate or chitosan. These polymers demonstrate high selectivity to water and considerable chemical and mechanical resistance, ensuring very good separation properties, especially for mixed matrix membranes [4,5]. To further improve their performance, membranes can be filled with modifiers, e.g., metal oxides [6,7,8,9], metal–organic frameworks (MOFs) [10,11,12,13], graphene and graphene oxide [14,15,16], zeolites [17,18,19,20] and organic fillers [21]. The fillers can enhance the process of separation by several different mechanisms. For instance, when Cheng et al. [10] studied the transport properties through alginate membranes filled with Fe^III^-HMOF-5 in the process of ethanol dehydration via pervaporation, they noticed that the hollow structure of Fe^III^-HMOF-5 and the properties of Fe^III^ promoted the diffusion of water molecules. On the other hand, the superior performance of graphene-loaded sodium alginate nanocomposite membranes for the dehydration of isopropanol was associated with a low packing density of the filler, typically observed for the lowest concentration of graphene (2 wt.%). Interestingly, two or more fillers simultaneously added to the membrane might exhibit a synergy effect that can affect the separation process. For instance, Nigiz et al. [20] compared the effectiveness of sodium alginate membranes filled with synthetic zeolite 4A and natural zeolite clinoptilolite (CL) in the process of PV ethanol–water mixture separation. The size of zeolites was found to have an explicit impact on the flux but not on the membrane’s selectivity. Incorporation of both CL and 4A zeolites into an alginate matrix showed a synergy effect, allowing the membrane performance to be enhanced. A recent approach suggests the use of fillers exhibiting magnetic properties and this choice is supported by the fact that magnetized water is characterised by a decrease in specific heat and boiling point driving to the increase in evaporation [22,23]. In our previous works, we investigated alginate membranes filled with magnetite (Fe_3_O_4_) and hematite (Fe_2_O_3_) [24,25]. We noticed that the flux increased significantly and continuously with the increase in filler content and the highest flux was obtained for membranes filled with magnetite. A similar situation was also observed for the separation factor. This phenomenon may be explained by the fact that the separation properties of such membranes were improved not only by the creation of free volume into the polymer matrix but also as a consequence of magnetic properties having a great impact on the physical properties, stabilisation and permeation of water molecules [26,27]. The incorporation of magnetite into chitosan and alginate matrices has improved their selectivity, leading to the preferential passage of water rather than ethanol, based on the interactions among separated molecules and the magnetic field [22,23]. For all fillers, the crucial factor influencing separation effectiveness is their dispersibility in a polymer matrix [8]. Therefore, the major limitation of the use of magnetite is its tendency to form agglomerates [9,23].

Manganese dioxide, on the other hand, has been reported as one of the most attractive inorganic materials, possessing a great potential as a membrane filler due to its hydrophilicity and, consequently, better water uptake [28]. Depending on the allotropic form, manganese dioxide exhibits also paramagnetic or superparamagnetic properties. As observed in a previous study [29], manganese ion can replace the iron ion due to nearly similar atomic radius and it can affect the physicochemical properties of magnetite, that seems to be a valuable material for various applications, e.g., MRI contrast and adsorption of heavy metals [30,31,32,33]. Having this in mind, we decided to fabricate a composite alginate membrane filled with a mixture of manganese dioxide and magnetite, expecting that the presence of manganese dioxide as a co-filler would improve dispersion of magnetite and enhance membrane selectivity. Accordingly, this manuscript presents the preparation of mixed filler membranes, their physicochemical characterization and comparison of effectiveness in the process of ethanol dehydration via PV.

## 2. Experimental Procedure

### 2.1. Materials

Sodium alginate (1 wt.%; Brookfield viscosity, 350–550 mPa·s at 20 °C), potassium hydroxide (purity ≥85%), potassium permanganate (purity ≥98%), manganese(II) chloride tetrahydrate (purity ≥98%), iron(III) chloride hexahydrate (purity ≥97%), iron(II) chloride tetrahydrate (purity ≥98%) and ethanol (96%, extra pure) were obtained from Acros Organics. Calcium chloride (purity ≥96%) and ammonia solution (25 wt.% solution in water) were supplied by Avantor Performance. All chemicals were used as received without further purification.

### 2.2. Preparation of Investigated Fillers

Manganese dioxide was prepared based on Rosas’ method [34]. Firstly, the following three solutions were prepared: (1) 1.12 g of KOH was dissolved in 20 cm^3^ of distilled water, (2) 0.936 g of KMnO_4_ was dissolved in 30 cm^3^ of distilled water and (3) 1.97 g of MnCl_2_∙4H_2_O was dissolved in 10 cm^3^ of distilled water. Then, 14 cm^3^ of KOH solution (1) and 23 cm^3^ of potassium permanganate solution (2) were poured into a beaker containing 500 cm^3^ of distilled water and continuously stirred, obtaining a solution with a pH of 12.4. Furthermore, 7 cm^3^ of the manganese (II) chloride solution (3) was added dropwise within 5 min to the above mixture. The suspension of precipitated manganese dioxide was continuously agitated for a period of 30 min; later, it was filtered, washed with distilled water and air-dried.

In the case of magnetite, Khalafalla’s [35] protocol was applied. To synthesize magnetite particles, 12 g of iron(II) chloride tetrahydrate and 24 g of iron(III) chloride hexahydrate were dissolved in 50 cm^3^ of distilled water and slowly mixed with 50 cm^3^ of a concentrated (25 wt.%) ammonium hydroxide solution. The precipitate was decanted and washed twice with a 50 cm^3^ portion of a diluted (1.25 wt.%) ammonium hydroxide solution until the chloride ions were completely washed out. The obtained stable magnetic fluid was dried at 50 °C for 2 days and in a planetary ball mill to obtain particles of D50 ~650 nm.

The MnO_2_@Fe_3_O_4_ mixture filler was synthesized by dispersing 3.48 g of previously prepared fresh magnetite in a KOH/potassium permanganate solution used for the precipitation of manganese dioxide as described above [34]. The suspension was continuously stirred for 30 min. Later, the precipitate was separated from the solution using a magnet, washed with distilled water and dried in air.

### 2.3. Membrane Preparation

To prepare composite alginate membranes filled with manganese dioxide and a mixture of manganese dioxide with magnetite, 4.57 g sodium alginate was dissolved in 300 cm^3^ of distilled water and mixed with an appropriate portion of filler to obtain 2, 5, 7, 10, 12 and 15 wt.% concentration of filler in the membrane. To ensure the good dispersion of the filler, the mixture was stirred in an ultrasonic bath for 1–3 h. Immediately after preparation, the solution was poured into levelled Petri dishes and dried at 40 °C for 24–48 h. The dry membrane was cross-linked with a 2.5 wt.% CaCl_2_ solution for 2 h. Later, the cross-linking agent was removed and the membrane was washed three times with distilled water. The average thickness of the investigated membranes was in the range 25–30 ± 1.2 μm.

### 2.4. Physicochemical Characterization

The morphology of the membranes was characterized using scanning electron microscopy (SEM; Phenom Pro-X working at 15 kV). The degree of swelling (*DS*) of pristine and composite Alg membranes filled with manganese dioxide and manganese dioxide/magnetite mixed filler was determined using a sorption test. In this case, pieces of membrane were immersed in distilled water and the mass changes of the analysed samples were determined during 24 h using analytical balance. The degree of swelling (*DS*) was calculated using the following equation:

(1) $$DS=\frac{W_{wet}-W_{dry}}{W_{dry}}\cdot 100\;\left[\%\right]$$

where *W_wet_* and *W_dry_* are the mass of a wet and dry membrane, respectively.

The contact angles of dry membranes were measured using an MDA1300 Handheld Metal Microscope. Each measurement was done twice, immediately after dropping and after a period of 10 s.

### 2.5. Pervaporation Experiments

PV experiments were carried out using the experimental set-up described previously [9]. As a feed, 96 wt.% ethanol was used. The feed tank was pumped with a circulating pump directly to the feed side of a separation chamber. The permeate was collected in a cold trap cooled with liquid nitrogen. The low pressure was produced by a vacuum pump and controlled with a vacuum gauge. The retentate stream was turned back to the feed tank. The mass of collected permeate was determined at fixed time intervals and a corresponding permeation flux of component *i* (*J_i_*) was calculated using the following equation [36]:

(2) $$J_{i}=\frac{m_{i}}{A\cdot t}\left[\frac{g}{m^{2}\cdot s}\right]$$

where *m_i_* is the mass of component *i* in permeate (g), A is an effective membrane area (m^2^), *t* is permeation time (s).

The separation factor determines the practical ability to separate mixture components into permeate and retentate. For a binary mixture, it can be described using the formula [36]

(3) $$\alpha_{A/B}=\frac{y_{A}/y_{B}}{x_{A}/x_{B}}$$

where *x_A_* and *x_B_* are weight fractions of *A* and *B* components in the feed (wt.%), respectively and, *y_A_* and *y_B_* are weight fractions of *A* and *B* components in the permeate (wt.%), respectively.

The permeation coefficient determines the “speed” of permeating species passing through the membrane, under the influence of the difference of its pressure on the feed and permeate sides. The following formula is characterised by Fick’s law [36]:

(4) $$P_{i}=J_{i}\frac{l}{\Delta p}\left[Barrer\right]$$

where *J_i_* is a diffusion flux of component *i* $\left[\frac{cm^{3}{}_{STP}}{cm^{2}\cdot s}\right]$*, l* is the membrane’s thickness (cm), Δp is a difference between pressure on the feed and permeate side (cmHg).

The diffusion coefficient was estimated using the method described in thte literature [37], assuming that the analysed membranes were not empty before the measurement. It also considers the effect of the tubing length between the permeation cell and the traps. According to this method, the diffusion coefficient is calculated by the formula

(5) $$D=\frac{-l^{2}}{3L_{a}}$$

where $L_{a}$ is the effective total time lag calculated as follows: $L_{a}=L_{a}^{2}-6.5\cdot L_{a}^{1};\;L_{a}^{1}$ is a time lag related to the tubing (*s*)*;* $L_{a}^{2}$ is an asymptotic part of time lag (*s*); and *l* is the thickness of the membrane, (*cm*).

The solubility coefficient states the level of the penetrant’s sorption within the membrane and it is calculated using following equation [36]:

(6) $$S=\frac{P}{D}\;\left[\frac{cm^{3}{}_{STP}}{cm^{3}\cdot cmHg}\right]$$

where *P* is a permeability coefficient [Barrer] and D is a diffusion coefficient $\left[\frac{cm^{2}}{s}\right]$.

## 3. Results and Discussion

### 3.1. Membranes’ Morphology

**Figure 1:**
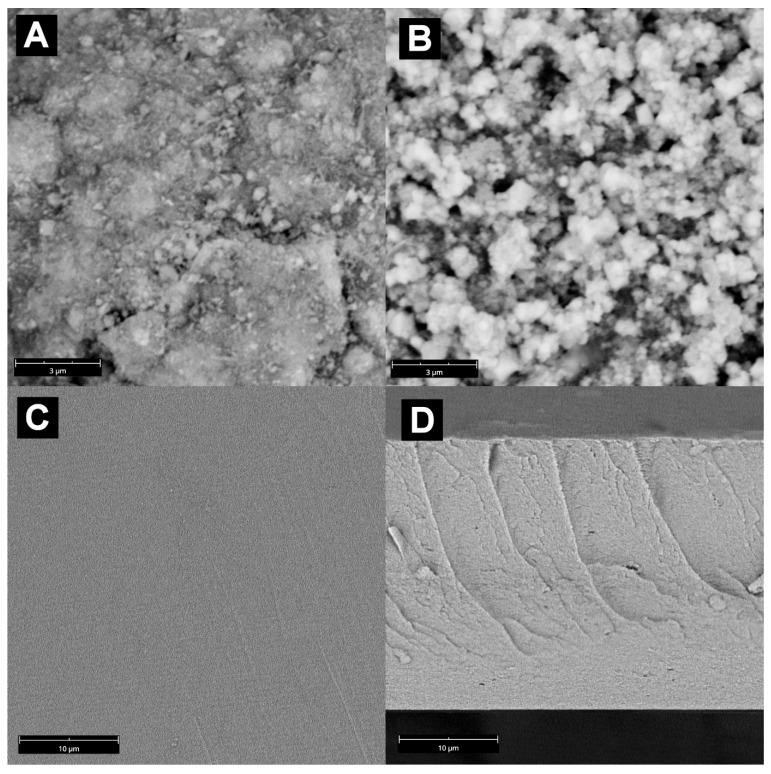
The SEM images of synthesized fillers. (**A**)—MnO_2_. (**B**)—mixed MnO_2_@Fe_3_O_4_ at 20 k magnification and pristine alginate membrane: (**C**)—surface; (**D**)—cross-section at 7 k magnification.

The SEM images of the synthesized MnO_2_ and MnO_2_@Fe_3_O_4_ mixed filler particles, pristine membrane and two type composite membranes loaded with MnO_2_ and mixed MnO_2_@Fe_3_O_4_ filler are presented in Figure 1. It can be observed that the MnO_2_ synthesized according to the procedure described in paragraph 2.2 consisted of small particles, with a diameter starting from ca. 100 nm, that were joint together and formed big agglomerates approaching 10 μm in size. The synthesis of the mixed MnO_2_@Fe_3_O_4_ filler allowed us to reduce the dimension of the particles, as a result of MnO_2_ crystallization on the surface of magnetite nanoparticles that induced the structure and direction of the growth of MnO_2_ crystals [29]. The morphology of MnO_2_@Fe_3_O_4_ was found to mimic a cauliflower-like structure, composed of about 200 nm grains that built up a bigger assembly with size in the range from 0.5 to 1.5 μm. The nucleation and crystallization of MnO_2_ on magnetite cores resulted in the formation of particles with a fractal geometry. Consequently, the synthesis of mixed MnO_2_@Fe_3_O_4_ oxides resulted in the control over the ability to agglomerate both MnO_2_ itself and strongly ferromagnetic Fe_3_O_4_. Because of the strong magnetic attractive force, Fe_3_O_4_ easily assembles and forms 5 μm or bigger agglomerates [38], which is the main drawback of the application of Fe_3_O_4_ as a filler in magnetic membranes. The addition of MnO_2_ to Fe_3_O_4_ allowed us to overcome the typical challenge of using the latter, that is, achieving proper dispersion and stabilization of Fe_3_O_4_ nanoparticles inside a polymer matrix.

**Figure 2:**
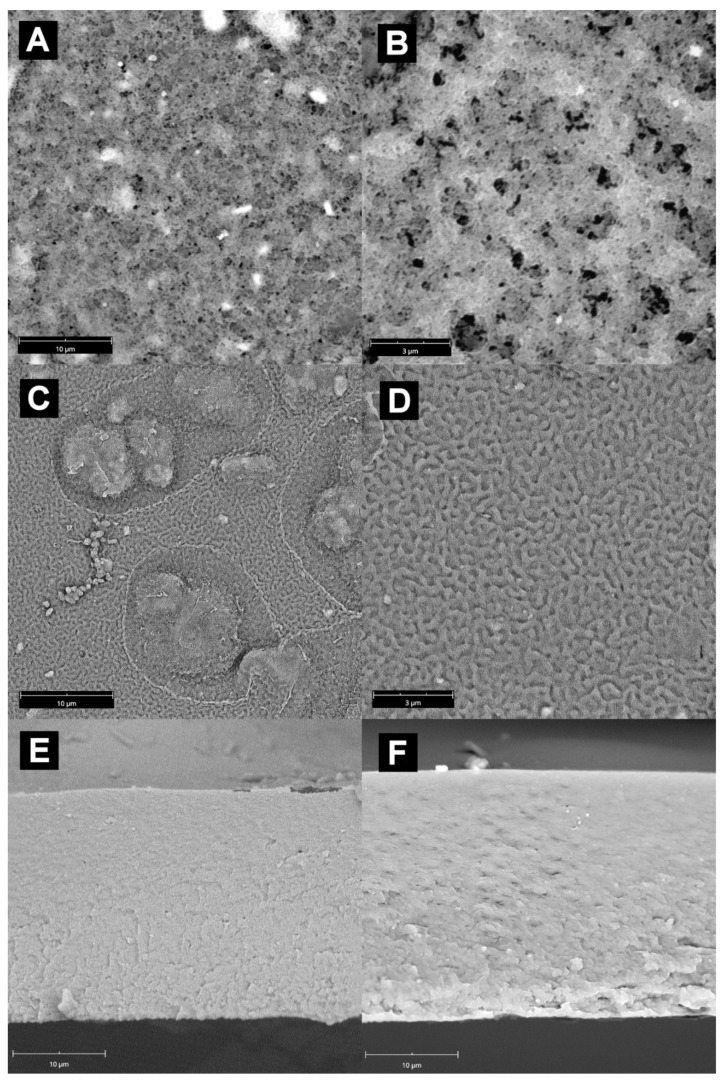
The SEM images of Alg–MnO_2_ membranes: (**A**,**B**)—membrane surface with 5 wt.% filler content, magnification 7 k and 20 k, respectively; (**C**,**D**)—membrane surface with 15 wt.% filler content, magnification 7 k and 20 k, respectively; (**E**,**F**)—cross-section of membrane with 5 wt.% and 15 wt.% filler content, respectively. Magnification 7 k.

**Figure 3:**
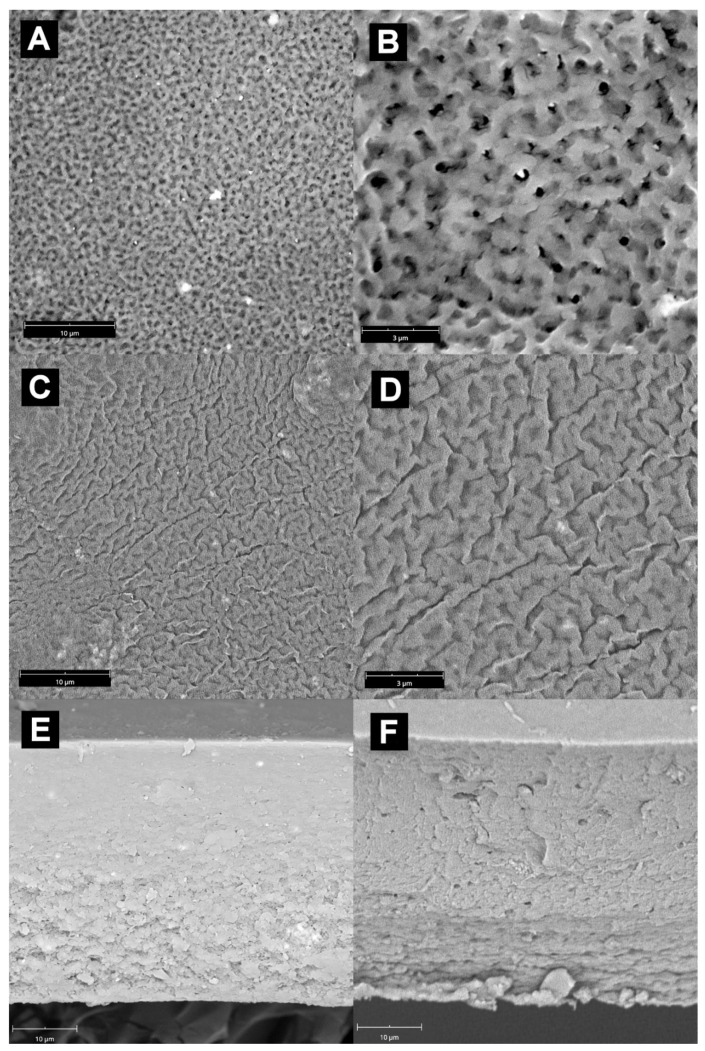
The SEM images of Alg– MnO_2_@Fe_3_O_4_ membranes: (**A**,**B**)—membrane surface with 5 wt.% filler content, magnification 7 k and 20 k, respectively; (**C**,**D**)—membrane surface with 15 wt.% filler content, magnification 7 k and 20 k, respectively; (**E**,**F**)—cross-section of membrane with 5 wt.% and 15 wt.% filler content, respectively. Magnification 7 k.

The images of surface and cross-sectional views of plain alginate crosslinked membrane show a typical, smooth structure of a dense polymer membrane with no voids indicating phase separation. A completely different structure of membranes containing 5 and 15 wt.% of both types of fillers was revelated by the SEM images presented in Figure 2 and Figure 3. As it can be seen, composite membranes were characterized by the phase separation induced by the presence of a filler, which propagated from the bottom edge to some extent along the membranes’ cross-section. Phase separation led to the formation of a highly corrugated, biomimicking 3D morphology of composite membranes. The developed structure depended both on the nature of filler and its loading in the membranes. In the case of the Alg–MnO_2_ membrane with 5 wt.% filler loading, fine sponge-like structures with numerous channels formed (Figure 2). This kind of structure is frequently observed in mesoporous silica [39]. The unbroken agglomerates of MnO_2_ in 5 wt.% loaded membranes were rare and the typical size of filler grains was about 2–4 μm. Increasing the filling degree of MnO_2_ up to 15 wt.% changed the surface morphology and promoted the development of fine and wavy walnut-like structures. Contrary to the membranes with lower amounts of filler, the higher amount of MnO_2_ resulted in the predominant presence of unbroken filler’s assemblies in the form of ca. 10 μm agglomerates. At a higher magnification, the smaller submicron particles of the MnO_2_ filler could also be observed. The boost in the extent to which the phase separation appeared in the membrane’s cross-section for the increase in filler content could be noticed. Phase separation was seen in the bottom part of membrane, which is evidence of the asymmetric distribution of a filler along the thickness of the membrane due to the sedimentation of heavy oxide particles.

The morphology of composite membranes containing mixed MnO_2_@Fe_3_O_4_ filler is similar to those that contain only MnO_2_, but these membranes are generally “coarser” (Figure 3); thus, for 5 wt.% content of mixed filler, the membrane also had a sponge-like structure, but the walls and connections were significantly thicker. Likewise, the structure of a membrane containing 15 wt.% of MnO_2_@Fe_3_O_4_ resembled the walnut surface, but with thicker walls and maze-like features.

The main difference between both types of fillers present in Alg–MnO_2_@Fe_3_O_4_ membranes was the lack of agglomeration of mixed filler, regardless of filler content. As assumed, the growth of MnO_2_ onto magnetite cores successfully overcame the tendency of Fe_3_O_4_ to agglomerate as a result of an attractive magnetic force. The presence of MnO_2_ layer diminished this attraction, so the size of filler particles dispersed inside the alginate matrix corresponded to the diameter of the synthesized MnO_2_@Fe_3_O_4_ mixed filler itself and was in the range 0.5–2 μm. The distribution of the mixed filler was also improved. It could be seen, from the cross-section, that the phase separation extented more significatively across the membrane thickness and, for 15 wt.% loading of MnO_2_@Fe_3_O_4_, was visible almost throughout the whole cross-section.

### 3.2. Pervaporation Performance

**Figure 4:**
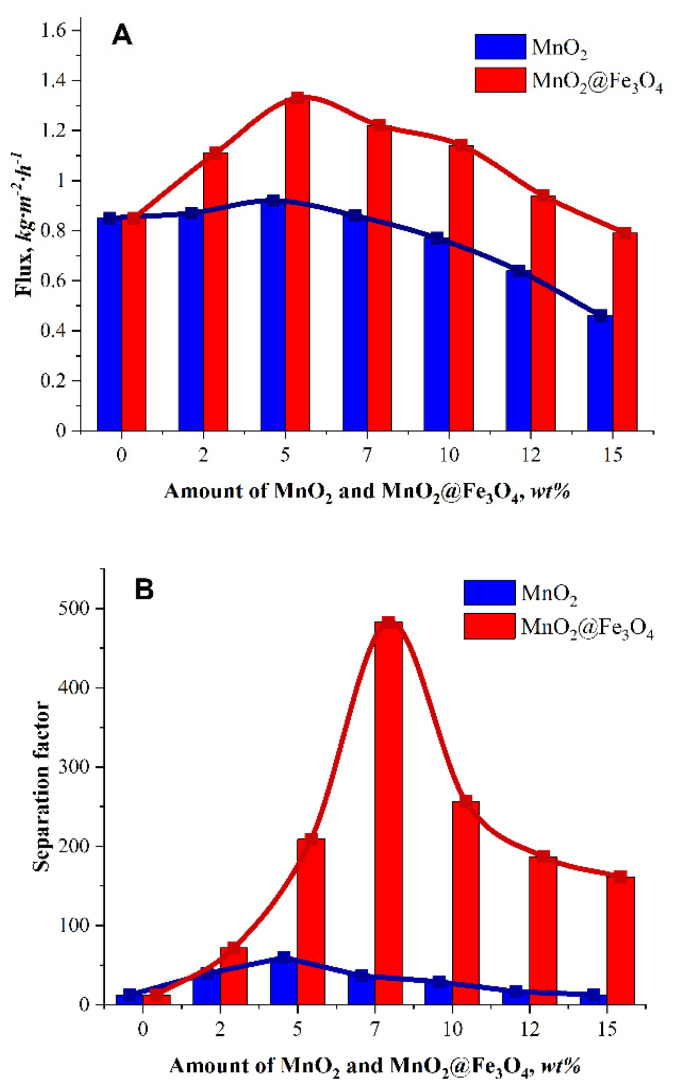
The dependence of total flux (**A**) and separation factor (**B**) on the amount of MnO_2_ (blue) or MnO_2_@Fe_3_O_4_ (red) fillers present in the membrane.

The collected pervaporation data derived from the mass and concentration of permeate over time of PV experimental runs (measurements) using Alg–MnO_2_ and Alg–MnO_2_@Fe_3_O_4_ membranes are shown in Figure 4. The effects of the fillers content on the total flux and separation factor for the dehydration of 96 vol% ethanol feed at 298 K are shown in Figure 4A,B, respectively. The results indicate that both the separation factor and flux increased with the increase in filler content up to 5 wt.%. However, the highest separation factor was observed at 5 wt.% for the MnO_2_ filler and at 7 wt.% for the MnO_2_@Fe_3_O_4_ mixed filler. The simultaneous increase in flux and separation factor shows that a trade-off effect between these two parameters can be overcome to a certain degree with either of the investigated composite alginate membranes. After exceeding the optimum loading of filler, the separation factor and flux were found to gradually decrease. A similar tendency in the behaviour of flux and separation factor was observed by Li et al. [40] in the study on isopropanol–water mixture PV separation with polyamide (PA) composite membranes prepared in a double interfacial polymerization of PA on a nanofibrous electrospun polyacrylonitrile fibre substrate. It was noticed that the excess concentration of trimesoyl chloride monomer in organic phase caused the formation of too-thick selective layer, leading to a decline in the molecular sieving efficiency and membrane stability and, consequently, a decrease in the separation factor and total flux. Comparing the fluxes and separation factors obtained for membranes with MnO_2_ and MnO_2_@Fe_3_O_4_ fillers, it can be noticed that the highest values were observed for the mixed MnO_2_@Fe_3_O_4_ filler. For this membrane, the flux was ca. 45% higher than that of the membrane with a neat MnO_2_ filler and the maximum value of separation factor equalled to 483, which is more than 8 times higher than that for MnO_2_.

**Figure 5:**
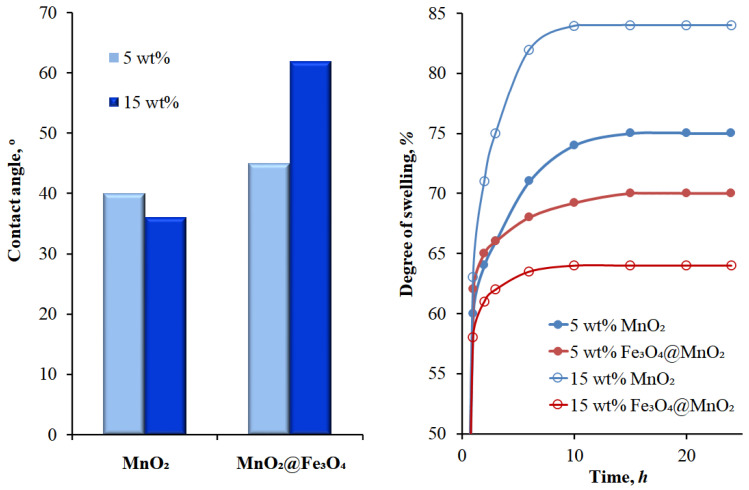
The changes in contact angle (**left panel**) and the kinetic of swelling (**right panel**) for alginate membranes filled with neat MnO_2_ and MnO_2_@Fe_3_O_4_ mixed fillers at 5 and 15 wt.%, respectively.

The difference in membrane hydrophilicity (Figure 5A,B) is one of the possible reasons for the distinctiveness of a membrane’s separation efficiency. The application of neat MnO_2_ as a filler promoted the membrane’s hydrophilicity to a higher extent than the presence of MnO_2_@Fe_3_O_4_. The contact angle of MnO_2_-filled membranes decreased for higher loading degrees of the filler (from 40° at 5 wt.% to 35° at 15 wt.%); further, this effect was accompanied with the increase in the degree of swelling (from 75 to 84%). The preparation of a mixed MnO_2_@Fe_3_O_4_ filler by crystallization of MnO_2_ on the surface of magnetite nanoparticles changed the hydrophilicity of the membranes containing this filler. Such membranes were less hydrophilic and this tendency was enhanced by the filler content; therefore, for these membranes, an increase in the contact angle (from 45° to 62°) and a decrease in the degree of swelling (from 70% to 64%) was found. The swelling equilibrium of alginate membranes was reached after ca. 15 h for membranes with 5 wt.% content of filler and a bit faster for higher-loaded membranes, i.e., ~10 h (Figure 5, right panel). Gimenes et al. [41] found that the increase in hydrophilicity by blending PVA with sericin extracted from silkworm cocoons caused excessive swelling of the membrane and resulted in a higher permeation flux, as well as a depressed separation factor. On the contrary, in our study, less hydrophilic membranes with the mixed MnO_2_@Fe_3_O_4_ filler exhibited noticeable higher values of total flux. The changes in the total flux and separation factor were also associated with the decrease in effective cross-section area at higher filler loadings and the enhanced magnetic character, mainly of the magnetite core, in the mixed filler that affected the membrane’s separation properties. When investigating the magnetic-field transfer of water molecules, Osuga and Tatsuoka [34] stated that the faster diffusion of water molecules in membranes with magnetic particles is related to the increase in the melting temperature of the magnetic field. A water molecule is composed of two hydrogen atoms and one oxygen atom with the centre of gravity located approximately on the negatively charged oxygen nucleus. Thermal excitation enables the rotation of positively charged hydrogens around the oxygen. Throughout the movement, a short electric current is generated that produces a Lorentz force, which causes water molecules to behave similarly to a diffusing molecule and make a random walk in the magnetic field—a phenomenon responsible for the accelerated water diffusion through the membranes. The interactions of the magnetic field with water has already been studied both experimentally and theoretically by many researchers in the past [22,28,34,42,43,44,45]. The major challenge seems to be to achieve the smooth, uniform distribution of a mixed filler within a membrane material. When the current results are compared with the results obtained earlier for the composite membranes filled only with neat magnetite [24], one can see that the achieved separation factor was similar to the one described for Alg–MnO_2_ (SF = 60 at 15 wt.% of magnetite). A distinct difference was observed in the case of total flux, where this was equal to ca. 13 kg·m^−2^·h^−1^ and was found to continue to grow with a magnetite loading, but with a simultaneous decrease in separation efficiency, which was related to the notable separation between polymer matrix and a filler. Neat magnetite is a strong ferromagnetic material and, as was shown in [38], forms huge agglomerates (even up to 20 μm) inside a polymer matrix. The application of a mixed filler allowed us to control the size of the filler particles and overcome the tendency towards its agglomeration. The pronounced deterioration in the separation effectiveness at 10 wt.% and higher MnO_2_@Fe_3_O_4_ loading is evidence of the gradual distortion of the membrane structure.

**Figure 6:**
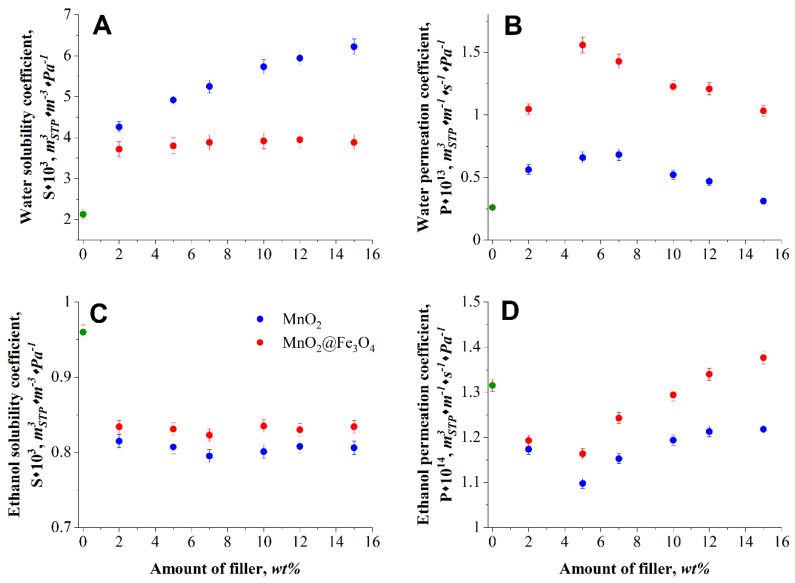
Calculated transport coefficient for alginate membranes filled with MnO_2_ and MnO_2_@Fe_3_O_4_: panel (**A**)—water solubility coefficients; (**B**)—water permeation coefficients; (**C**)—ethanol solubility coefficients; (**D**)—ethanol permeation coefficients.

The determined dual-sorption transport parameters were consistent with the hydrophilicity results determined from the contact angles and swelling measurements (Figure 6). The water solubility coefficient in alginate membrane increased with the amount of filler and was more prominent for more hydrophilic Alg–MnO_2_ membranes and at the highest filler loading. For both investigated membranes, the water permeation coefficient reached a maximum value in the range 5–7 wt.% filler content and was greater in the case of membranes that contained the mixed MnO_2_@Fe_3_O_4_ filler. With the same range of filler loading, the function of permeation coefficient of ethanol versus filler content had a minimum. The solubility of ethanol was confined to some extent in the presence of both type of fillers and this effect was slightly larger due to the higher hydrophilicity of MnO_2_. Thus, it can be concluded that the low solubility of ethanol in the membrane is one of the factors determining the effectiveness of ethanol transport through the membranes containing MnO_2_ and mixed MnO_2_@Fe_3_O_4_ fillers. Considering the water solubility and permeation coefficient, one can state that the diffusion process plays a main role in water transport for this type of membranes. As demonstrated by Osuga and Tatsuoka [42], in the presence of magnetic field, the diffusion of water molecules can be accelerated.

The comparison of the data concerning the composite membranes containing different types of metal oxide used for pervaporation dehydration of ethanol available in the literature with our results are presented in Table 1.

As it can be seen, the composite membranes filled with metal oxides have been widely investigated for the separation of alcohol–water solutions through PV. The preparation of composite alginate membranes with MnO_2_ filler increased the separation efficiency of ethanol–water mixture, making them better than the most membranes collected in Table 1. Moreover, further modification of alginate membrane through the replacement of MnO_2_ oxide with a mixed MnO_2_@Fe_3_O_4_ filler led to the significant improvement of separation selectivity. This membrane performed with a separation factor equal to 483 and moderately high total flux—1.22 kg∙m^−2^·h^−1^. Only a thin membrane prepared on the porous PAN support by Zhao et al. [37] showed better efficiency. However, it can be noted that this result was achieved at an elevated temperature in the PV process and, under similar process conditions, the pervaporation performance was much worse—the flux was ca. ten times lower and the separation factor was more than 4 times lower if compared with our Alg–MnO_2_@Fe_3_O_4_ membrane.

## 4. Conclusions

Alginate composite membranes filled with MnO_2_ and mixed MnO_2_@Fe_3_O_4_ particles were successfully applied in pervaporative dehydration of ethanol. The significant difference between these two fillers consisted in the stronger magnetic properties of the latter, borne by the magnetite core of the mixed MnO_2_@Fe_3_O_4_ filler that accelerated the diffusion of water molecules in the membrane. The results show that the membranes containing MnO_2_ filler were more hydrophilic than the membranes with the mixed MnO_2_@Fe_3_O_4_ filler. In the investigated membranes, changes in the separation factor and flux had similar trends, i.e., growth or decrease in both parameters were observed; thus, there was an optimum filler loading when both quantities reached their maximum. Consequently, the best results were obtained for the alginate membrane filled with 7 wt.% of mixed MnO_2_@Fe_3_O_4_ filler, for which the separation factor and flux equalled to 483 and 1.22 kg·m^−2^·h^−1^, respectively. This result was achieved thanks to the magnetic properties of the filler and its structure, that allowed filler agglomeration to be avoided, as well as providing a better control of particle size distribution and nearly uniform dispersion in the polymer matrix.

## Figures and Tables

**Table 1:** The comparison of investigated membranes with other composite membranes reported in the literature, filled with various metal oxide particles, used in ethanol dehydration via pervaporation.

Polymer Matrix	Filler	Flux kg·m^−2^·h^−1^	Separation Factor	Temperature °C	References
SA/PAN	8 wt.% PAA/Fe_3_O_4_	1.63 **0.121**	1044.0 **101.1**	77 **30**	[7]
CHIT	TiO_2_	0.34	196.0	80	[46]
CHIT	Fe_3_O_4_	-	3.3	25	[9]
SA	Fe_3_O_4_	1.38	58.8	25	[24]
SA	Ag_2_O	0.81	33.8	25	[8]
SA	TiO_2_	0.85	33.3	25	[8]
SA	Cr_2_O_3_	1.09	28.3	25	[8]
SA	ZnO	1.28	30.3	25	[8]
SA	5 wt.% MnO_2_	0.91	59.8	25	Present work
SA	7 wt.% MnO_2_@Fe_3_O_4_	**1.22**	**483.0**	**25**	Present work

## Data Availability

Not applicable.
